# Compositional and Physicochemical Characterization of Cashew Apple Bagasse (*Anacardium occidentale* L.) as a Potential Lignocellulosic Feedstock

**DOI:** 10.1021/acsomega.5c11610

**Published:** 2025-12-10

**Authors:** Tiago Linhares Cruz Tabosa Barroso, Luiz Eduardo Nochi Castro, Vanessa Cosme Ferreira, Felipe Sanchez Bragagnolo, Leda Maria Saragiotto Colpini, Mauricio Ariel Rostagno, Rosana Goldbeck, Tânia Forster-Carneiro

**Affiliations:** † Faculdade de Engenharia de Alimentos (FEA), 28132 Universidade Estadual de Campinas (UNICAMP), Rua Monteiro Lobato, 80, Campinas 13083-862, São Paulo, Brazil; ‡ Laboratório Multidisciplinar de Alimentos e Saúde (LabMAS), Faculdade de Ciências Aplicadas (FCA), Universidade Estadual de Campinas (UNICAMP), Rua Pedro Zaccaria, 1300, Limeira 13484-350, São Paulo, Brazil; § Federal University of Parana (UFPR), Jandaia do Sul 86900-000, Paraná, Brazil

## Abstract

Cashew apple bagasse (*Anacardium occidentale* L.) is an agricultural byproduct that has been minimally studied. Still, it offers many potential uses in the food, pharmaceutical, and energy industries. This study provides a comprehensive characterization of this biomass, including its physicochemical, structural, thermal, and phytochemical properties, to clarify its functional and technological applications. The material showed a high dietary fiber content (45.01% NDF), reduced sugars (34.22 mg g^–1^), and protein content (11.96%), along with low water activity (0.58), which supports microbiological stability. UPLC–PDA–MS analysis identified several bioactive compounds—including ferulic acid, chlorogenic acid, myricetin, quercetin, and anacardic acids—that are known for their antioxidant, antimicrobial, and anti-inflammatory effects. The lipid fraction was mainly composed of oleic acid (ω-9, 62.71%) with a balanced ω-6/ω-3 ratio (∼1.6:1), indicating high nutritional value and oxidative stability. Structural and morphological analyses confirmed a dense fibrous matrix rich in cellulose and hemicellulose, while thermal analyses showed stability up to 360 °C, supporting its use in thermochemical or extrusion-based processes. Overall, these findings suggest that cashew apple bagasse is a promising lignocellulosic feedstock for use in functional foods, nutraceuticals, and biopolymer production, promoting circular and sustainable industrial practices. However, its successful performance in such applications will rely on future research focused on process optimization and product validation.

## Graphical abstract

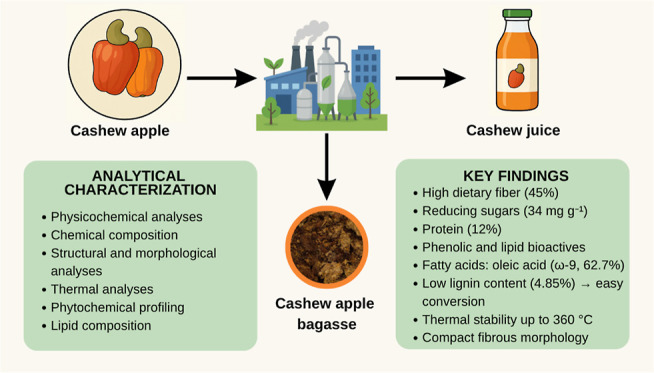

## Introduction

The cashew apple (*Anacardium occidentale* L.) is a tropical pseudofruit with significant economic and social value, especially in countries like Ivory Coast, India, and Vietnam, which are among the top producers worldwide.^1,2^ It is widely enjoyed as juice, alcoholic beverages, syrups, soft drinks, and preserves.^3,4^ However, during the industrial processing of cashew apples, the main byproduct produced is bagasse, a residue material often discarded or underutilized, despite its high potential for various uses.^1,5^

Cashew apple bagasse is a fibrous byproduct generated in large quantities during the processing of cashew apples. It has a high content of dietary fiber, fermentable sugars, pectin, and bioactive compounds such as polyphenols, making it a promising biomass for applications in the food, pharmaceutical, cosmetic, packaging, and energy industries.^6–8^ In alignment with circular economy principles, the valorization of agro-industrial residues such as cashew apple bagasse offers a sustainable pathway to produce high-value products, thereby mitigating environmental impacts and fostering economic development. Cashew apple bagasse, comprising approximately 20% of the cashew apple’s weight, is rich in bioactive compounds, including phenolics, flavonoids, and dietary fibers, which can be harnessed for various applications. For instance, studies have demonstrated that pectin can be extracted with yields up to 23.37% using pressurized liquid extraction (PLE), surpassing conventional techniques.^9^ Additionally, bagasse serves as a substrate for microbial fermentation, producing bioethanol, citric acid, and probiotic beverages.^10^ These value-added products not only reduce waste but also support local economies by creating new markets and job opportunities. Despite its potential, the systematic characterization and integration of cashew apple bagasse into valorization frameworks remain underexplored, necessitating further research to optimize extraction processes and develop sustainable utilization pathways.^5,11^

To promote the rational and efficient use of this biomass, it is essential to conduct comprehensive characterization of the biomass. Understanding its physical–chemical, structural, and functional properties is vital for grasping its thermal behavior, composition, morphology, presence of functional groups, and other important features.^12^ These parameters are decisive in guiding utilization processes. For example, in the bakery industry, the development of functional food or pharmaceutical products and raw materials with low moisture and high microbiological stability is prioritized;^13,14^ in nutritional supplements or functional beverages, the presence of soluble and bioavailable bioactive compounds is important.^15,16^ The high fiber, protein, and pectin content also favor its application in the production of biodegradable packaging,^17,18^ while the fermentable sugar content is essential for fermentation processes aimed at the production of biofuels or enzymes.^19,20^ However, there are still a few studies that integrate these multiple potentialities into a biorefinery model applied to cashew apple bagasse. This research aims to fill this gap with a comprehensive characterization that can address potential biomass utilization failures.

Therefore, possessing a comprehensive knowledge of the properties of biomass is essential for guiding its proper and efficient utilization in industrial processes. Characterization studies of agro-industrial residues, such as cashew apple bagasse, facilitate the identification of their potential for producing bioproducts and supporting sustainable technological solutions. In this context, this study aims to thoroughly characterize cashew apple bagasse, thereby promoting its rational application across various industrial sectors through data-driven decision-making.

## Materials and Methods

### Sample Preparation

The cashew apple bagasse used in the study came from the pulp industry in Ceará, Brazil. The fruit was harvested between September and October 2023, during which all postharvest practices were followed. The fruit was immediately processed in industrial depulpers, and the residual bagasse was stored in polypropylene plastic containers at temperatures below −20 °C. The cashew apple bagasse was dried in a forced air oven at 60 °C for 48 h. Afterward, the material was ground using a knife mill and sifted through a 600 mm sieve. Finally, it was stored at −18 °C until the characterization analyses were performed.

### Sample Characterization

#### Physical Parameters

The pH was measured using a digital pH meter (IonLab, model THS-3E, USA), which had been calibrated beforehand with buffers at pH 4.0 and 7.0. The titratable acidity was determined by the titrimetric method.^21^ The hygroscopicity was adapted from the method of Cai et al.,^22^ evaluating temperatures of 10, 25, and 40 °C and relative humidity (RH) with saturated solutions of LiCl (11% RH), K_2_CO_3_ (43% RH), NaCl (75% RH), and K_2_SO_4_ (98% RH). The water activity was measured at 25 °C by using Aqualab equipment (Decagon, USA). The water displacement method, based on Archimedes’ principle, was employed to determine the apparent density. The CIELaB color was evaluated with a portable colorimeter (CM-600d, Konica Minolta, Japan). All measurements were made in triplicate (*n* = 3).

#### Chemical Parameters

The determinations according to AOAC^21^ of moisture (925.10), ash content (923.03), and protein (992.23) were performed. Lipid content was determined by the Bligh–Dyer method.^23^ The determination of total and reducing sugars was performed by the Somogyi–Nelson method.^24^ The Weende method^25^ was used to determine the crude fiber. Neutral detergent fiber and acid detergent were measured using the Van Soest method.^26^ The values of cellulose, hemicellulose, and lignin were determined by the Van Soest and Wine method.^27^ The chemical oxygen demand (COD) was determined using the method outlined in the Standard Methods for the Examination of Water and Wastewater.^28^ In this method, organic matter is oxidized by potassium dichromate under strongly acidic conditions. The amount of dichromate reduced during the reaction is then measured spectrophotometrically (at 610 nm), and the COD is expressed as the oxygen equivalent of the oxidizable material.^26^ The elemental analysis was performed in a CHNS–O element analyzer (Thermo Fisher Scientific Inc., The Netherlands).

#### Morphological, Structural, and Thermal Parameters

The morphology was examined with a scanning electron microscope (Hitachi TM4000Plus, Japan), operating under vacuum and an electron beam accelerating at 5 kV. Morphological porosity was determined from scanning electron microscopy (SEM) micrographs using ImageJ software, with the adapted method from AlMarzooqi et al.^29^ Initially, the images were converted to 8 bit. Then, a threshold binarization process (Threshold, Moments method) was applied to segment the porous phase from the solid matrix. The final porosity was quantified as the area fraction of the pores. Quantification was performed in three distinct regions of the image to calculate the mean and standard deviation. Structural analysis was carried out using Fourier transform infrared (FTIR) spectroscopy (BRUKER TENSOR 37, Germany), covering the range from 650 to 4000 cm^–1^, with a 4 cm^–1^ resolution and 128 scans. Thermogravimetric analysis (TGA) was conducted using a simultaneous thermal analyzer (STA 6000, PerkinElmer, USA), from 50 to 900 °C. The heating rate was set at 20 °C/min with a nitrogen flow of 20 mL per minute. The sample, weighing 10 mg, was placed in an alumina crucible. Differential scanning calorimetry (DSC) measurements were carried out with a DSC1 instrument (Mettler Toledo, Switzerland). Tests were performed at 25 ± 3 °C using N_2_ as the carrier gas at 50 mL/min, spanning a temperature range 0–250 °C with a heating rate of 10 °C per minute.

#### UPLC–PDA–MS Analysis

Four extraction techniques were used for UPLC–PDA–MS analysis to identify compounds from the cashew apple bagasse. These included (A) 100% methanol (MeOH) with ultrasound-assisted extraction (UAE), (B) a 50% MeOH–water mixture with UAE, (C) pure water with UAE, and (D) pure water via pressurized liquid extraction (PLE) at 15 MPA and 150 °C. UAE was performed using an ultrasonic bath (Solidsteel, model SSBu 3.8 L, Brazil) for 15 min at 25 °C, with an ultrasonic power of 160 W. All methods started with 10 g of sample and 100 mL of solvent. Extracts were diluted 5-fold in water, filtered through a 0.22 μm nylon filter, and injected into a UPLC–PDA–MS system (Acquity, Waters, USA). The separation was performed using the method described by Jimenez Moreno et al.^30^ For compound identification, UPLC–PDA–MS data were compared with UV and MS spectra of compounds previously reported in cashew apple bagasse, enabling tentative identification of potential constituents.^31^

#### Lipid Composition by Gas Chromatography

The oil previously extracted using the Bligh–Dyer method was subjected to the procedure described in Brito et al.’s^32^ study. The fatty acids in the oil are derivatized into their methyl esters (FAMEs). A gas chromatograph connected to a single quadrupole mass spectrometer (GC–MS; Agilent 7890A GC system, Agilent Technologies Inc., Santa Clara, CA, USA) was employed to evaluate the fatty acids. A fused silica capillary column (J&W DB-23, Agilent Inc., Santa Clara, CA, USA; 60 m × 0.25 mm) was utilized for the separation process. The mass spectra were used to confirm the identification of the fatty acids by comparison with those from the National Institute of Standards and Technology (NIST) spectral library (NIST, Gaithersburg, MD, USA) and by comparing the retention times with those of the standard compounds (FAME 37 component mix, Supelco 47885). Each analysis was conducted in duplicate. The proportion of each component was determined by dividing its peak area by that of the internal standard. Using a calibration curve created with methyl nonadecanoate, the findings were expressed as a percentage or in μg mL^–1^.

### Statistical Analysis

The results were reported as means with standard deviations with all measurements performed in duplicate or triplicate.

## Results and Discussion

### Physical Parameter Determination

The physical parameters used to characterize the cashew apple bagasse are listed in Table 1. The pH value was acidic (4.5), which is typical for tropical fruits and aligns with the range commonly reported in the literature for cashew juice, from 3.2 to 4.7,^33^ as well as for the pomace (4.5–4.8).^34,35^ The pH maintenance in its pomace is also attributed to the purely physical process of extracting the cashew pulp. Additionally, titratable acidity indicates organic acids such as tartaric, citric, fumaric, oxalic, and ascorbic acids, which have previously been reported in cashew.^36^ This parameter directly impacts the stability of the material regarding storage, microbiology, and potential technological applications of cashew apple bagasse. Organic acids have demonstrated considerable biotechnological interest, whether for use in food, pharmaceuticals, cosmetics, resins, metal cleaning, or as a sequestrant in wastewater treatment processes.^37^

**Table 1:** Physicochemical Properties of the Cashew Apple Bagasse

parameter	value
pH	4.5 ± 0.03
titratable acidity	0.0419 ± 0.0008 citric acid/g wet sample
water activity	0.5837 ± 0.0025
density	1.2519 ± 0.0006 g mL^–1^

With a water activity (Aw) around 0.4, the product can be classified as a low-moisture food, which is crucial to determine its storage conditions and safety measures.^38^ This parameter is important because it is possible to understand the material’s susceptibility to microbiological degradation since the reduced availability of water inhibits the growth of bacteria, yeasts, and molds.^39^ This data also influence the chemical and enzymatic activity of the sample. High water activity (Aw) values are linked to increased lipid oxidation and hydrolysis, as well as the breakdown of compounds like sugars and phenolics.^38^ When cashew apple bagasse is considered as a matrix for extracting biocompounds, Aw plays an important role in several capacities. When the material has a low Aw content, it may suffer from compaction by cellular hardening, thus preventing the accessibility of the extractive solvent.^40^

On the other hand, high levels of Aw induce swelling of the plant matrix, which promotes cell rupture and subsequent release of compounds.^41^ It is imperative to recognize that this parameter also influences the selection of potential extractive solvents, such as hydroalcoholic mixtures; the ethanol/water ratio can facilitate or obstruct sample accessibility, depending on the Aw levels.^42^ Furthermore, Aw is a crucial parameter to assess when adding cashew apple bagasse to food formulations because of its impact on the microbial stability and product quality. For example, lipid oxidation occurs below Aw = 0.30, and the Maillard reaction peaks at Aw = 0.65.^43^ Moisture content can affect the texture and stability of the final product, ultimately influencing the consumer acceptance and shelf life.

In this context, Maciel et al.’s^44^ study proposed a process for commercializing freeze-dried cashew apple bagasse, beginning with five pretreatment cycles involving washing and pressing to reduce the bagasse’s acidity and fruity flavor, followed by freeze-drying. The same study also evaluated the formulation of various foods with freeze-dried cashew apple fiber, including “coxinha”, kibbeh, chickpea sausage, and pea-based mini burgers, obtaining analogous products with excellent flavors and visual appearance.

The density of the residue can be considered high when compared to other biomasses, such as sorghum, soy, and tobacco, which are between 0.22 and 0.45 g mL^–1^.^45^ A more compact material, such as cashew apple bagasse, is interesting from a logistical point of view, as it reduces the volume occupied both in distribution centers and in direct applications, since fermentation or extraction reactors will occupy less volume. However, this same characteristic can hinder the diffusion of solvents in extractive processes, which can be reversed by applying more modern techniques that allow greater solvent penetration, such as pressurized liquids.^46^

Table 2 shows a significant increase in water absorption in cold, wet environments during hygroscopicity analysis, indicating that the cashew apple bagasse is sensitive to humidity. At 10 °C and 75% RH, the moisture absorbed was 25.74 g 100 g^–1^, but at 25 °C, the values were more consistent (11.16– 15.74 g 100 g^–1^), indicating better resistance to absorption at temperature. Absorption increased to 21.00 g 100 g^–1^ at 40 °C and 98% RH, emphasizing the need for precautions under hot and humid conditions. These results emphasize the need to regulate storage conditions and indicate the bagasse’s potential in formulations that depend on water retention management.

**Table 2:** Hygroscopicity Analysis of the Cashew Apple Bagasse

room temperature (°C)	relative humidity (%)	hygroscopicity (g of moisture 100 g–1 of dry sample)
10	11	12.47 ± 0.66
	43	20.77 ± 0.22
	75	25.74 ± 0.43
	98	22.28 ± 0.63
25	11	11.16 ± 0.10
	43	15.74 ± 0.17
	75	15.74 ± 0.12
	98	15.31 ± 0.12
40	11	9.89 ± 0.01
	43	14.82 ± 0.11
	75	17.75 ± 0.46
	98	21.00 ± 0.57

Figure 1 illustrates the cashew apple bagasse after drying and the artificial color generated from the CIELAB color parameters (L* = 31.35 ± 2.22; a* = 13.38 ± 0.03; b* = 41.27 ± 2.25; C* = 43.39 ± 2.14). The sample exhibits a dark orange-brown hue. Brown tones are strongly linked to the caramelization processes of the sugars present and the occurrence of phenolic compounds.^47^ The color of vegetable biomass varies significantly depending on its application. If applied directly to food formulations, it must undergo sensory evaluation to determine whether a notable color change in the final product could impact consumer acceptance.

**Figure 1:**
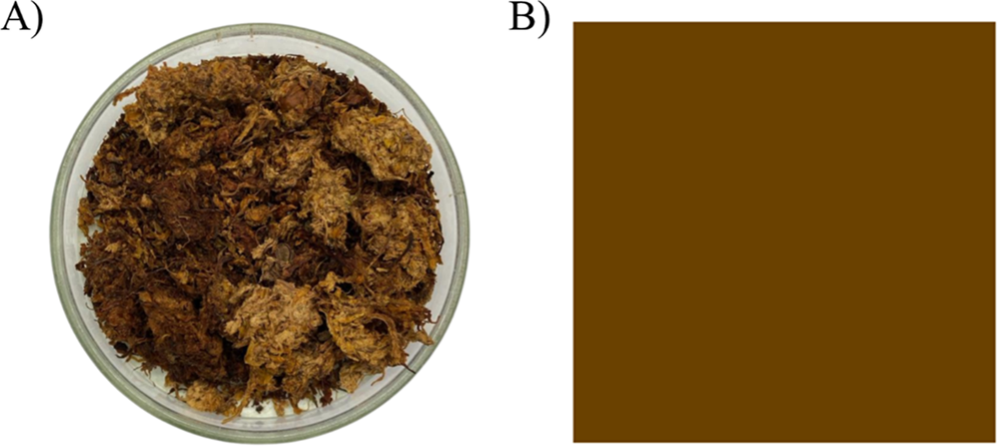
Cashew apple bagasse (A) material after drying and (B) coloration obtained in CIELAB color space.

### Chemical Parameter Determination

Table 3 shows the results of the chemical characterization of the cashew apple bagasse. The moisture and ash contents obtained in this study are consistent with values previously reported in the literature. For instance, Abdoulaye et al.^48^ reported 10.61% moisture and 3.4% ash, while Rodrigues et al.^49^ found 9.71% moisture and a slightly higher ash content of 4.45%. These variations are common and can be attributed to several factors that influence the composition of cashew apples, such as geographical location, soil and climate conditions, and rainfall patterns. Additionally, the amount of ash directly impacts the energy production efficiency. The ash generated during and after heating can also affect the sizing of the energy system. As Bhatt et al.^50^ pointed out, in thermoelectric plants, as ash content rises from 6% to 75%, boiler area needs to increase by 69%, and overall efficiency drops to 77%, showing significant adverse effects on performance. Also, the increased generation of residual ash after the process poses significant environmental risks when incorrect disposal occurs.^51^

**Table 3:** Chemical Parameters of the Cashew Apple Bagasse

parameter	value
moisture	10.97 ± 0.05 (%, w/w)
ash	2.18 ± 0.01 (%, w/w)
protein	11.96 ± 0.06 (%, w/w)
lipid	2.65 ± 0.07 (%, w/w)
total sugar	39.74 ± 0.36 (mg g^–1^)
reducing sugar	34.22 ± 0.30 (mg g^–1^)
crude fiber	12.62 ± 0.40 (%, w/w)
neutral-detergent fiber	45.01 ± 0.99 (%, w/w)
acid-detergent fiber	17.55 ± 0.19 (%, w/w)
cellulose	12.70 ± 0.19 (%, w/w)
lignin	4.85 ± 0.31 (%, w/w)
hemicellulose	27.46 ± 1.04 (%, w/w)
COD	125.00 ± 0.98 (g O_2_ L^–1^)
carbon	45.37 (%, w/w)
hydrogen	6.06 (%, w/w)
nitrogen	1.02 (%, w/w)
oxygen	47.56 (%, w/w)

The vegetable protein scene is booming, driven by the search for new sources of protein and technological treatments that enhance its potential and digestibility. When compared to established traditional sources, cashew apple bagasse does not perform as well as soybeans (40%)^52^ and peas (33.9%).^53^ However, in developing a biorefinery centered around cashew apple bagasse, its comprehensive utilization must be evaluated at both a technical–economical level and the processes needed for protein extraction. Compared to other values in the literature, Nguyen et al.^54^ found 12.50%, which closely aligns with the 12.75% found by Salehi et al.^55^ and the 11.56% reported by Freitas et al.^56^ Zie et al.^57^ studied a protein–polysaccharide complex, indicating that it can function effectively as a thickener, stabilizer, gelling agent, or emulsifier.

As a result, the lipid values reported in the literature vary significantly, with some studies indicating high values of 13.24%^58^ and others showing 4.10%.^48^ As previously mentioned, the low value observed in this study may be attributed to abiotic and biotic factors. The low lipid content found could be used in the creation of healthier foods. In this proposal, de Oliveira Silva et al.^59^ prepared churros dough with up to 10% substitution of traditional wheat flour with malt flour, resulting in a product that is both low in lipids and high in protein content. The sugar results indicate that most of the available sugars (total sugar), without applying a hydrolytic treatment to the biomass, are reducing sugars. The presence of reducing sugars is a potential factor in the evaluation of biomass for the fermentative process. As already present in other studies, cashew apple bagasse is usually applied in a pretreatment that allows the saccharification of its structure for various purposes, such as ethanol, hydrogen, and xylitol production.^60–62^

The high fiber content of the material provides promising potential for developing plant-based food products. Its fibrous structure can be used in making items like “paçoca”, meatballs,^63^ and croquettes.^64^ However, this structural complexity also requires attention when processes such as saccharification are involved, as it might reduce access. For example, the acid-detergent fiber fraction (17.55%) is less accessible during most hydrolysis steps,^65^ emphasizing the need to choose an appropriate pretreatment strategy.

The high quantity of hemicellulose indicates a source of xylose and functional oligosaccharides.^66^ The low concentration of lignin facilitates energy routes that are not necessary such as the very aggressive process. Silva et al.^61^ reported a process in which an alkaline pretreatment (hydrogen peroxide) was followed by an acid process under pressure (sulfuric acid, 1 atm; 121 °C), after which the material was inoculated with *Clostridium roseum*. This strategy enabled a biogas yield of approximately 12 mL L^–1^ h^–1^, comprising 72% hydrogen and 28% carbon dioxide.

The COD value found is high when compared to other sources of biomass in agroindustry. Wastewater from pig farming, breweries, and dairy farms has COD values of 18.707, 5.536, and 17.629 g O_2_ L^–1^, respectively.^67^ This parameter is crucial for understanding the material’s organic load and how significantly it affects environmental disposal without prior treatment. Additionally, it serves as a strong indicator of the potential use of conversion in biogas for anaerobic digestion.^68^ Finally, the elemental composition demonstrates that cashew apple bagasse is composed of carbon and oxygen, very similar to other lignocellulosic residues.^69^ This also reinforces potential by utilizing energetic biomass due to its carbon portion in the synthesizing adsorbent.

### Morphological, Structural, and Thermal Parameters

Figure 2A shows a micrograph of the cashew apple bagasse, depicting the surface. This surface is dense and compact. This structure is significant due to the presence of high fibers in the material, which are organized in overlapping layers. The porosity of cashew apple bagasse obtained in this study (42.06 ± 3.32) is notably lower than that of other commonly studied lignocellulosic residues, such as rice husk (67.86%), rice straw (77.24%), sugar cane bagasse (84.26%), and cotton stalk (59.37%).^70^ This difference indicates that cashew apple bagasse has a more compact structure with fewer interconnected voids, which can directly affect its liquid and gas absorption properties and solute diffusion capacity. The noticeably low porosity formation is typical in biomass with a high concentration of fibers or lignin. The quality can limit the diffusion of solvents or hydrophobic agents, affecting the yields in extraction or hydrolysis. This reinforces the use of the pretreatments when the intention is to access sugar in the matrix. However, this fibrous morphology can be advantageous when applying materials, such as films, cryogels, and composites, where it is beneficial for the structure’s integrity to have greater mechanical resistance.^71^ The same characteristics can also be positively applied in food formulations, contributing to the structural properties, increasing fiber content in foods, or in applications aimed at controlling the release of bioactive compounds during digestion.^72^ However, care must be taken regarding the decrease in water retention and palatability in hydrated food systems. In a systematic review of fiber modification, Gan et al.^73^ addressed that modification (physical, chemical, biological, or a combination of them) can be achieved through increasing soluble fiber, enhancing functional properties (such as water retention and cholesterol adsorption), improving technological properties (e.g., solubility and particle size), enhancing digestibility, and promoting bioactivity.

**Figure 2:**
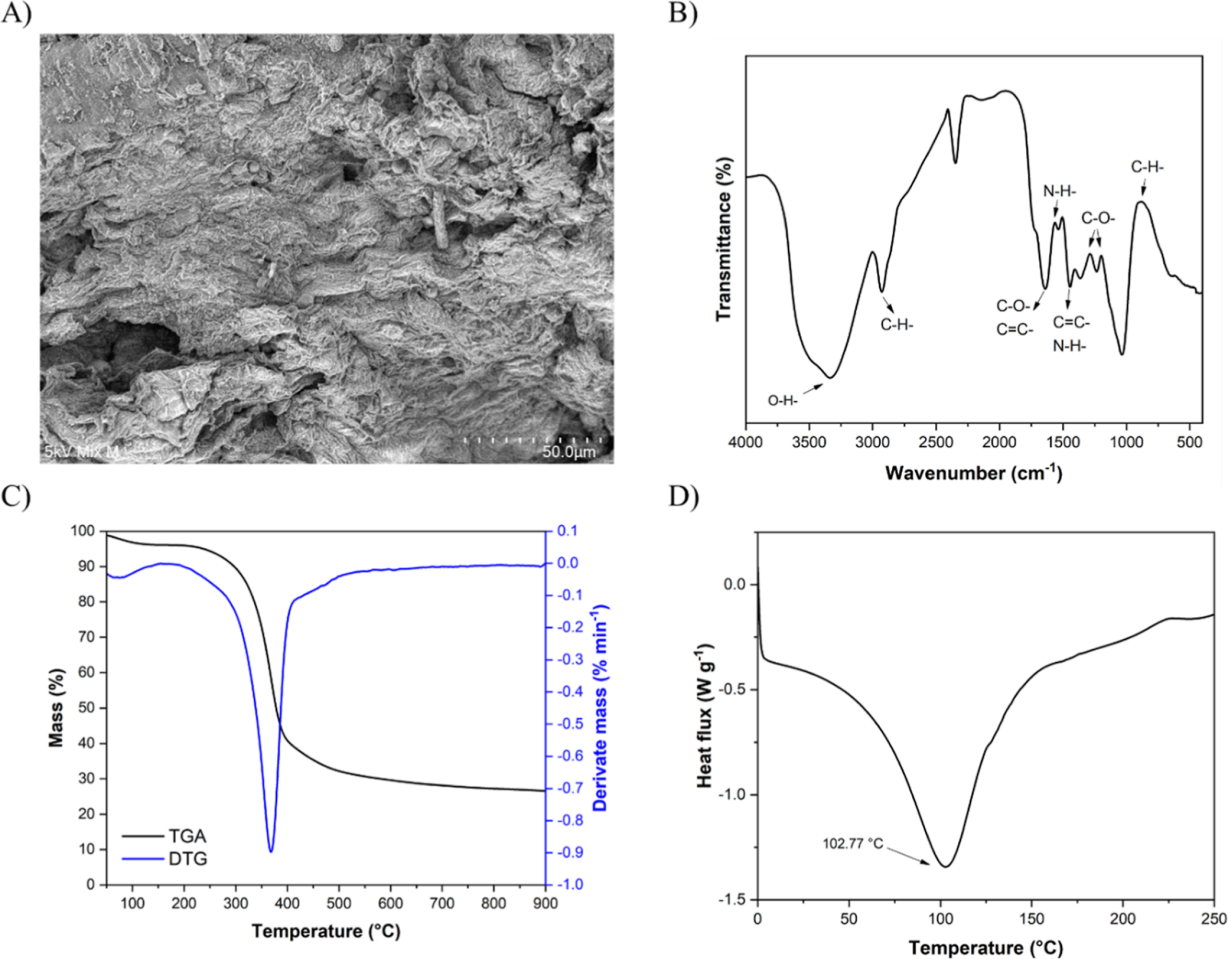
Morphological, structural, and thermal parameters of cashew apple bagasse. SEM image at 500× magnification (A), FTIR spectrum (B), TGA curve (C), and DSC analysis curve (D).

Figure 2B shows the FTIR spectrum of cashew apple bagasse along with the material’s compositional functional groups. The first band at 3335 cm^–1^ is commonly found in lignocellulosic biomass, corresponding to the O–H stretching of cellulose.^74^ A similar band can be observed around 2925 cm^–1^, which is associated with the aliphatic C–H stretching of alkane and alkene groups present in lignin.^75^ Additionally, a band around 1650 cm^–1^ corresponding to C=C or C–O stretching vibrations was observed, which can be attributed to lignin or aromatic groups.^74,76^ The identification of amines in cashew apple bagasse is linked to N–H stretching around 1550 cm^–1^.^77^ A band observed around 1460 cm^–1^ is attributed to C–H stretching vibrations typical of alkanes and aldehydes, whereas the C–O stretching vibrations of aromatic esters appear between 1300 and 1350 cm^–1^.^78^ Finally, a C–H stretch around 800–900 cm^–1^ is due to the vibrations from the deformation of CH bonds associated with aromatic rings.^79^ Knowledge of the FTIR spectrum of biomasses enables a range of applications, including chemical characterization and the identification of functional groups, as well as estimating potential interactions with polymers and additives.^80^ Understanding these functional groups also helps us identify active sites naturally present in biomass for adsorption studies, for example.^80^

Figure 2C displays the TGA and DTG profiles of the cashew apple bagasse. The initial weight loss seen around 100 °C corresponds to the evaporation of physically adsorbed and bound water.^74^ This aligns with the low water activity previously reported (Aw = 0.58). The main degradation stage, with a prominent DTG peak near 360 °C, relates to the breakdown of hemicellulose and cellulose fractions. This stage accounts for a substantial mass loss (approximately 65%), indicating the dominance of structural carbohydrates in cashew apple bagasse, as confirmed by its chemical composition (45.01% NDF; 27.46% hemicellulose; 12.70% cellulose). Hemicellulose degradation generally begins between 175 °C and 330 °C, while cellulose decomposes from 330 °C to 370 °C.^81^ The overlap of these events results in a broad, intense peak around 360 °C. Above 370 °C, the rate of decomposition declines sharply, suggesting the formation of stable carbon-rich residues mainly derived from lignin and minerals.^81^ The remaining mass at 900 °C (about 25%) indicates incomplete lignin degradation and ash minerals, consistent with the ash content of 2.18% (Table 3). Cashew apple bagasse is thermally stable up to approximately 300 °C, making it suitable for thermal or extrusion processes, such as biochar production, thermoplastics, or encapsulating matrices. Its high carbon residue further supports its potential in activated carbon or adsorbent manufacturing, highlighting its versatility as a lignocellulosic feedstock.

The thermal analysis by DSC (Figure 2D) of cashew apple bagasse revealed an endothermic peak at 102.77 °C, accompanied by an enthalpy of 277.45 J g^–1^. DSC analysis helps to determine whether the reaction during biomass pyrolysis is endothermic or exothermic. When biomass is used in these processes, this previously obtained information allows for a more accurate estimate of the amount of heat required for the process.^82^ The thermal peak observed can be partly attributed to the removal of water retained within the biomass matrix, a phenomenon frequently observed in lignocellulosic materials, as reported for cambuci residue,^30^ rice husk, barley, and corn cob.^74^ The presence of water bound to hydrophilic structures, such as pectins and hemicelluloses, requires energy to be released, contributing to the detected endothermic signal. In addition to dehydration, the melting of other structural components of the biomass, mainly soluble carbohydrates, may also contribute to this peak.^82^ From an energy perspective, the occurrence of an endothermic peak indicates that when using cashew apple bagasse in pyrolysis processes an initial amount of energy will be required before the thermal transformations begin. On the other hand, in the field of materials, a high endothermic enthalpy suggests that the biomass has a good heat absorption capacity, which can be beneficial in applications that require thermal stability or thermal energy storage.^83^

### UPLC–PDA–MS Analysis

The extracts produced were subjected to UPLC–PDA–MS analysis to infer possible candidates from each extract. Based on previous literature reports on metabolites identified in cashew apple bagasse, tentative identifications for the compounds listed in Table 4 were proposed. Compound identification was carried out by using a cross-validation approach that combined three independent results: UV spectra, *m*/*z* values, and comparisons with previously published data in the literature. In forthcoming studies, authentic standards for the compounds of most significant interest will be employed to confirm these identifications and enable accurate quantification using methods such as NMR and QToF-MS.

**Table 4:** UPLC–PDA–MS Analyzed the Tentative Identification of Compounds in Extracts Obtained from Cashew Apple Bagasse^a^

retention time (min)	name of candidates	*m*/*z*	wavelength (nm)	fraction
0.34	ferulic acid	193.14 [M – H]^−^	255, 356	C
2.68	dihydrophaseic acid hexoside	443.37 [M – H]^−^		A, B, C
3.22	myricetin-*O*-hexoside	479.01 [M – H]^−^	264, 358	A, B, D
3.39	myricetin	319.13 [M + H]^+^	254, 354	B
3.42	myricetin-*O*-rhamnoside	463.21 [M – H]^−^	254, 365	A, B, C, D
3.52	chlorogenic acid	353.19 [M – H]^−^	253, 358	C
3.69	quercetin	303.15 [M + H]^+^	263, 347	B, D
3.72	quercetin-*O*-rhamnoside	447.27 [M – H]^−^	280	A, B, C
5.15	trihydroxy-octadecenoic acid	329.25[M – H]^−^		A, B, C, D
9.04	(15:3)-anacardic acid	341.48 [M – H]^−^	244, 310	A, C
9.44	(15:2)-anacardic acid	343.42 [M – H]^−^	244, 310	A

a Legend: 100% MeOH by UAE (A), 50% MeOH:50% H_2_O by UAE (B), 100% H_2_O by UAE (C), and 100% H_2_O by PLE at 15 MPa and 150 °C (D).

The analysis revealed a complex phytochemical profile with significant antioxidant, bioactive, and therapeutic potential.^8^ Specifically, the compound classes flavonoids (such as myricetin and quercetin) and phenolic acids (including ferulic and chlorogenic acids), as well as an exclusive cashew compound (anacardic acid).^8^ Solvent choice and extraction method greatly affect the yields and types of compounds extracted. This is evident in the different compounds obtained using the proposed methods A–D (see legend in Table 3). This study did not aim for exhaustive extraction or extraction-based methods; instead, it focused on a more exploratory approach. However, one can see the influence in the diffusive effects of the compounds, which are influenced by polarity phenomena, access a lignocellulosic structure, or even the thermal decomposition of the biocompounds.

Ferulic and chlorogenic acids are known phenolic compounds. Ferulic acid is a potent antioxidant, antiapoptotic, and antiplatelet agent.^84^ Chlorogenic acid, beyond its antioxidant action, has a role in glycemic modulation and liver and kidney protection.^85^ Myricetin has been studied and indicates antiviral action^86^ and quercetin’s neuroprotective action.^87^ Trihydroxy-octadecenoic acid has applications in the industries of resins, waxes, lubricants, cosmetics, and antimicrobial action.^88^ Dihydrophaseic acid hexoside is a metabolite of abscisic acid, a phytohormone involved in the plant’s stress response.^89^ Finally, the anacardic acids found are exclusive to cashews and have antibacterial, antitumor, and antioxidant properties and a strong capacity for metal chelation ions.^90^ It is important to note that these bioactivities are reported for the pure compounds in the literature and were not directly evaluated in the cashew apple bagasse extracts obtained in this study. Overall, the results indicate that cashew apple bagasse is a promising source of diverse bioactive compounds. While the findings highlight potential for applications in functional foods or nutraceuticals, further studies are needed to confirm these effects in the extracts themselves. In general, cashew apple bagasse has demonstrated a broad source of biocompounds that could potentially be extracted. The variety of bioactive compounds offers an opportunity to incorporate them into functional foods. Existing studies with cashew apple bagasse show an effect on modulating blood cholesterol,^91^ preventing oxidation reactions,^92^ and the potential for developing antidiabetic foods and nutraceuticals.^93^

The graph compares the effectiveness of different extraction methods and solvents in recovering compounds, with the height of the points presumably indicating the yield (Figure 3). The analysis shows that, in the UAE method, the hydroalcoholic mixture (B: 50% MeOH: 50% H_2_O) proved to be the most efficient, outperforming both pure methanol (A) and pure water (C), suggesting that intermediate polarity is ideal for extracting the broadest range of compounds of interest. Conversely, when comparing extraction with pure water, the use of the PLE technique (D) resulted in a significantly higher yield than UAE (C), highlighting the power of PLE as a highly effective “green” solvent. In instances where selectivity is observed in the extraction treatment for the analyte, certain behaviors are evident. Treatment C was the sole method capable of extracting ferulic and chlorogenic acids; treatment B was effective for myricetin; and treatment D was suitable for the (15:2)-anacardic acid.

**Figure 3:**
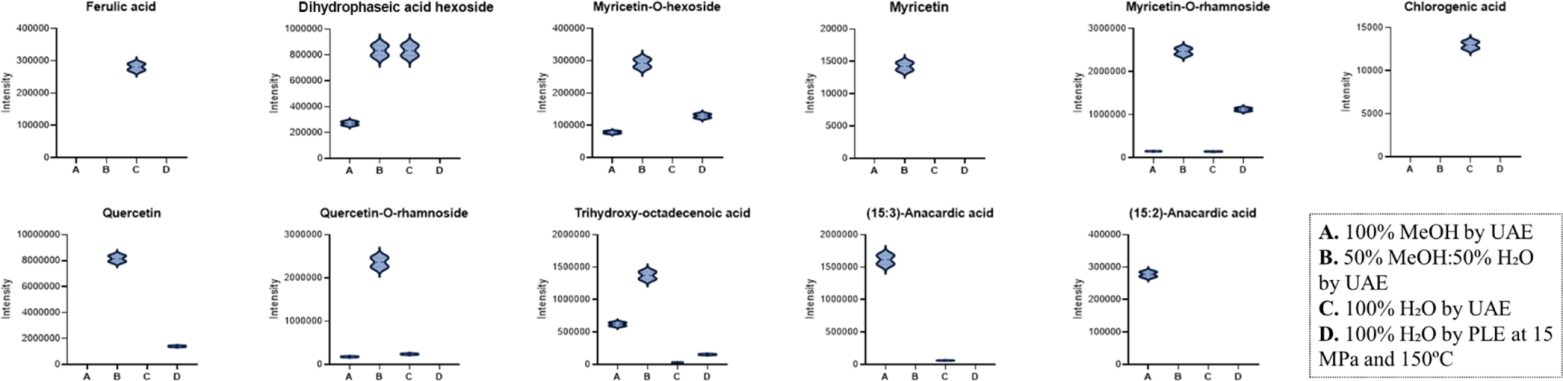
Intensity profiles of bioactive compounds detected by UPLC–PDA–MS in extracts obtained by 100% MeOH by UAE (A), 50% MeOH: 50% H_2_O by UAE (B), 100% H_2_O by UAE (C), and 100% H_2_O by PLE at 15 MPa and 150 °C (D).

### Lipid Composition

Table 5 presents the lipid profile of the oil extracted from the cashew apple bagasse. There is a strong predominance of monounsaturated fatty acids (MUFAs), particularly oleic acid (C18:1 *n*-9). From a nutritional perspective, this profile is desirable in vegetable oils because it is linked to beneficial effects on cardiovascular health and enhances oxidative stability.^94^ Palmitic acid (C16:0) stands out as the predominant saturated fatty acid (SFA). It ranks among the most abundant fatty acids in nature, found in plants, animals, algae, fungi, and yeasts, and is heavily incorporated into the human diet.^95^ While it remains unclear whether its consumption is detrimental, there is a recommendation to reduce intake or choose oils with lower palmitic acid concentrations.^95,96^ Additionally, polyunsaturated fatty acids (PUFAs) comprise 6.4%, such as linoleic (C18:2 *n* – 6) and α-linolenic (C18:3 *n* – 3) acids, both essential for the human diet.^97^ The balanced ratio of ω-6 to ω-3 (∼1.6:1) is noteworthy, as diets with higher ω-6 proportions can lead to inflammatory imbalances.^97^

**Table 5:** Lipid Profile of Oil Obtained from Cashew Apple Bagasse

**Fatty acid**		**%**
lauric acid	C12:0	0.33 ± 0.08
myristic acid	C14:0	0.29 ± 0.06
myristoleic acid	C14:1 *n* – 5	0.25 ± 0.05
palmitic acid	C16:0	19.31 ± 3.73
palmitoleic acid	C16:1 *n* – 7	1.64 ± 0.05
heptadecanoic acid	C17:0	0.20 ± 0.02
heptadecenoic acid	C17:1 *n* – 7	0.14 ± 0.03
stearic acid	C18:0	4.08 ± 0.19
oleic acid	C18:1 (n9)	62.71 ± 4.52
linoleic acid (LNA)	C18:2 (n6)	3.95 ± 0.34
α-linolenic acid (ALA)	C18:3 (n3)	2.49 ± 0.08
arachidic acid	C20:0	0.86 ± 0.14
eicosenoic acid (gondoic)	C20:1 (n9)	0.77 ± 1.08
behenic acid	C22:0	1.50 ± 0.12
erucic acid	C22:1 (n9)	0.31 ± 0.01
lignoceric acid	C24:0	1.19 ± 0.08

In the literature, only one other record of the lipid profile of cashew apple bagasse oil has been documented. The results obtained by Kouassi et al.^98^ identified nine fatty acids, with oleic acid as the major component at 64.10–64.69%, followed by palmitic acid at 19.36–20.77%, which is similar to the findings of this study. However, a lower amount of PUFAs was noted, with ω-3 and ω-6 each ranging from 1.80 to 2.29%, which could indicate oxidation occurring during processing or storage. The lower PUFA values reported may stem from oxidation during Soxhlet extraction with hexane (7 h at 60 °C), which involves prolonged heating and exposure to air. Such conditions involve prolonged heating and exposure to air, which can promote oxidation of polyunsaturated fatty acids. In contrast, in this study, lipids were extracted using the Bligh and Dyer cold method under gentle conditions, reducing PUFA oxidation and likely resulting in the higher yield observed. Consequently, the evaluated oil exhibits a nutritionally appealing profile. It holds potential for functional and technological applications in both the food and cosmetics industries, particularly due to its stable and healthful composition.

## Conclusions

Through comprehensive characterization, we can understand the biomass’s characteristics and indicate possible routes for its use. Applications in the fields of bioenergy and fermentation can be explored due to the sugars present in the matrix and its COD in anaerobic bioprocesses. The low lignin content (4.85%) is advantageous, as it facilitates hydrolytic conversion and reduces the need for harsh chemical treatments, supporting more sustainable processing strategies. Additionally, as confirmed by UPLC–PDA–MS, cashew apple bagasse has a significant phytochemical profile, including flavonoids, phenolic compounds, and anacardic acids. These compounds possess antioxidant, antimicrobial, anti-inflammatory, and neuroprotective properties, making extracts of cashew apple bagasse appealing for functional foods, nutraceuticals, and cosmetic applications. Cashew apple bagasse contains a lipid fraction with high levels of oleic acid (ω-9) (62.71%) and a balanced ω-6/ω-3 ratio (∼1.6:1), which is desirable for food and cosmetic formulations due to its cardiovascular benefits and oxidative stability.

In terms of technology, cashew apple bagasse can be effectively used in food formulations, especially for fiber-rich and plant-based product development. Its composition contains substantial amounts of crude fiber (12.62%) and protein (11.96%), enabling its use in plant-based formulations. Additionally, it possesses favorable morphological characteristics, including a compact fibrous surface with a low porosity, as revealed by SEM analysis. These attributes indicate the potential for the production of biopolymers, films, and cryogels, particularly in applications requiring structural integrity and mechanical resistance. Lastly, given its high carbon (45.37%) and oxygen (47.56%) contents, CAB is also suitable for thermochemical conversion into activated carbon or biochar, with uses in water purification, soil remediation, and adsorption systems. The elevated COD further underscores the necessity of proper waste management and supports the case for recovering energy through anaerobic digestion. Overall, cashew apple bagasse stands as a valuable agro-industrial byproduct with broad applicability in the bioenergy, food, chemical, and environmental sectors. Future research aiming to optimize compound extraction from cashew apple bagasse could benefit from incorporating environmental evaluation metrics, such as EcoScale, Green Star, and AGREE, to enhance the sustainability assessment of these processes.

## Data Availability

The corresponding author can provide the data upon reasonable request.
